# Lifetime exercise activity and breast cancer risk among post-menopausal women

**DOI:** 10.1038/sj.bjc.6690610

**Published:** 1999-08

**Authors:** C L Carpenter, R K Ross, A Paganini-Hill, L Bernstein

**Affiliations:** Department of Preventive Medicine, University of Southern California School of Medicine, USC/Norris Comprehensive Cancer Center, 1441 Eastlake Ave., Los Angeles, CA, USA

**Keywords:** breast neoplasms, exercise, weight gain, post-menopausal women

## Abstract

Lifetime exercise activity has been linked to breast cancer risk among young women. However, no study has specifically evaluated whether lifetime exercise activity is related to the breast cancer risk of post-menopausal women. We conducted a population-based case-control study of post-menopausal white women (1123 newly diagnosed cases and 904 healthy controls) aged 55–64 who lived in Los Angeles County, California, USA to evaluate this relationship. Although neither exercise activity from menarche to age 40 years, nor exercise after age 40 separately predicted breast cancer risk, risk was lower among women who had exercised each week for at least 17.6 MET-hours (metabolic equivalent of energy expenditure multiplied by hours of activity) since menarche than among inactive women (odds ratio (OR) = 0.55; 95% confidence interval (CI) 0.37–0.83). Exercise activity was not protective for women who gained considerable (> 17%) weight during adulthood. However, among women with more stable weight, breast cancer risk was substantially reduced for those who consistently exercised at high levels throughout their lifetime (OR = 0.42; 95% CI 0.24–0.75), those who exercised more than 4 h per week for at least 12 years (OR = 0.59; 95% CI 0.40–0.88), and those who exercised vigorously (24.5 MET-hours per week) during the most recent 10 years (OR = 0.52; 95% CI 0.32–0.85). Strenuous exercise appears to reduce breast cancer risk among post-menopausal women who do not gain sizable amounts of weight during adulthood. © 1999 Cancer Research Campaign


*British Journal of Cancer* (1999) **80**(11), 1852–1858
© 1999 Cancer Research Campaign
Article no. bjoc.1999.0610

### Lifetime exercise activity and breast cancer risk among post-menopausal women

**CL Carpenter, RK Ross, A Paganini-Hill and L Bernstein**

Department of Preventive Medicine, University of Southern California School of Medicine, USC/Norris Comprehensive Cancer Center, 1441 Eastlake Ave., MS-44, Los Angeles, CA, USA

**Summary** Lifetime exercise activity has been linked to breast cancer risk among young women. However, no study has specifically evaluated whether lifetime exercise activity is related to the breast cancer risk of post-menopausal women. We conducted a population-based case-control study of post-menopausal white women (1123 newly diagnosed cases and 904 healthy controls) aged 55–64 who lived in Los Angeles County, California, USA to evaluate this relationship. Although neither exercise activity from menarche to age 40 years, nor exercise after age 40 separately predicted breast cancer risk, risk was lower among women who had exercised each week for at least 17.6 MET-hours (metabolic equivalent of energy expenditure multiplied by hours of activity) since menarche than among inactive women (odds ratio (OR) = 0.55; 95% confidence interval (CI) 0.37–0.83). Exercise activity was not protective for women who gained considerable (> 17%) weight during adulthood. However, among women with more stable weight, breast cancer risk was substantially reduced for those who consistently exercised at high levels throughout their lifetime (OR = 0.42; 95% CI 0.24–0.75), those who exercised more than 4 h per week for at least 12 years (OR = 0.59; 95% CI 0.40–0.88), and those who exercised vigorously (24.5 MET-hours per week) during the most recent 10 years (OR = 0.52; 95% CI 0.32–0.85). Strenuous exercise appears to reduce breast cancer risk among post-menopausal women who do not gain sizable amounts of weight during adulthood.

**Keywords:** breast neoplasms; exercise; weight gain; post-menopausal women

Strenuous physical activity, known to influence ovarian hormone production, is a potentially modifiable factor that could lead to a reduction in breast cancer risk. Many studies of exercise and breast cancer risk have been conducted on premenopausal women (Bernstein et al, 1994; Chen et al, 1997; Gammon et al, 1998), and on pre- and post-menopausal women (Frisch et al, 1985; Albanes et al, 1989; Vihko et al, 1992; Dosemeci et al, 1993; Dorgan et al, 1994; Mittendorf et al, 1995; D'Avanzo et al, 1996; Coogan et al 1997; Thune et al, 1997; Mezzetti et al, 1998; Rockhill et al, 1998), and one exclusively among post-menopausal women (McTiernan et al, 1996).

Obesity and weight gain are important risk factors for post-menopausal breast cancer (Hunter and Willett, 1993; Huang et al, 1997). Strenuous exercise is associated with weight loss (Williamson et al, 1993), and consequently, physical activity, in addition to influencing ovarian hormone production during the reproductive years, may reduce post-menopausal breast cancer risk through the promotion of a lean body.

Information on exercise activities throughout women's lives is necessary to assess the relative importance of different potential biological effects of exercise. Although we previously examined lifetime history of exercise activity in relation to premenopausal breast cancer risk (Bernstein et al, 1994), to date no epidemiological study has evaluated this relationship among post-menopausal women. The present study examines this relation to determine whether the association varies by ages at which a woman engages in exercise activity, and weight change during adulthood.

*Received 20 October 1998*
*Revised 11 January 1999*
*Accepted 1 February 1999*

*Correspondence to:* L Bernstein

### MATERIALS AND METHODS

### Subjects

All white (including Hispanic) English-speaking female residents of Los Angeles County who were between the ages of 55 and 64 years at diagnosis, and born in the USA, Canada, or Western Europe, were eligible to participate in this study. A total of 2373 eligible breast cancer case patients identified by the University of Southern California Cancer Surveillance Program, the population-based cancer registry for Los Angeles County, were diagnosed with primary invasive or in situ breast cancer between 1 March 1987 and 31 December 1989.

We interviewed 1579 eligible patients (67%). We were unable to interview the remaining patients, 230 (10%) of which were too ill or had died; we could not locate 17 (1%); physicians denied us permission to contact 128 (5%); and 419 (18%) refused to participate.

One neighbourhood control subject was individually matched to 1506 interviewed case patients on birthdate (within 36 months) and race (hispanic, other white). To identify each neighbourhood control subject, we utilized a predefined walk pattern for the neighbourhood where the case patient lived at the time of her diagnosis. The median number of housing units approached per case was 25.

We obtained complete walk-pattern censuses of neighbourhoods for 636 case patients. For the remaining 870 breast cancer patients, we were denied access to at least one housing unit (median = 4) in the walk pattern. For 1205 breast cancer patients, the first identified eligible control subject participated. For 227 others, the second eligible control subject participated after the first refused; for 74 others, three or more eligible control subjects were identified.

Study procedures to protect human subjects were approved by the University of Southern California Institutional Review Board, in accord with assurances approved by the US Department of Health and Human Services. Each subject provided informed consent.

### Collection of data

In-person interviews for each case-control pair were generally conducted by the same female interviewer. We obtained lifetime reproductive, oral contraceptive, hormone replacement therapy and physical exercise histories for each participant. We recorded information up to a reference date (12 months prior to the date of the patient's diagnosis for each case and her matched control). We considered a woman to have a first-degree family history of breast cancer if her mother, sister or daughter had been diagnosed with breast cancer. We recorded self-reported height and weight at age 18 and at the reference date. Body mass was estimated by Quetelet's index (weight in kilograms divided by height in metres squared). We created a variable for per cent change in weight between age 18 and the reference date. Women who lost or had no weight change were designated the referent category and the remaining women were divided into three groups according to whether they were below the 50th percentile value, below the 75th percentile, or above the 75th percentile of control subjects.

We collected information on physical exercise activity in which women regularly participated for at least 2 h per week. We recorded the type of activity, ages started and stopped, and hours per week spent exercising. For each year of life, we computed total hours per week of all exercise activities, allowing for seasonal activities. We assigned metabolic equivalents of energy expenditure (MET) scores to each activity (Ainsworth et al, 1993), multiplied the score by hours per week engaged in that activity, and summed the MET-hour values across all activities for each year of a subject's life. Eighteen MET-hours of activity is equivalent to 3 h of hard vigorous exercise (MET = 6) such as jogging, field hockey and aerobics (Ainsworth et al, 1993).

We constructed two risk periods to assess exercise activity: menarche through age 39 (premenopausal period) and age 40 to the reference age (perimenopausal and post-menopausal periods). Annual MET-hours per week reported for each risk period were summed and divided by total years in that period. Cut-points were created for these two variables based on the joint distribution of average MET-hours per week in the two periods. We also used these cutpoints to evaluate lifetime exercise activity (since menarche). In all analyses, the referent group was women who were inactive during the relevant risk period. We assessed risk according to the total years each woman exercised at least 4 h per week, as this was an important marker in our study of younger women (Bernstein et al, 1994). We also examined risk in relation to the average MET-hours per week in the 10 years before each woman's reference date, and in the 10-year period following menarche.

### Analysis of data

We limited analyses to post-menopausal women with known ages at menopause and complete covariate information (1165 case patients, 1169 control subjects) after excluding subjects who were premenopausal (30 cases, 20 controls), who had unknown menopausal status (one control), who never menstruated (one control), who had a hysterectomy without bilateral oophorectomy prior to their last menstrual period (306 cases, 267 controls), who had a surgical menopause before age 30 (31 cases, 22 controls), who had a natural menopause before age 35 (seven cases, seven controls), who took oral contraceptives after menopause (31 cases, 15 controls), who were missing information on hormone replacement therapy (six cases, three controls), or who were missing measurements on weight or height (three cases, one control). We recreated matching strata for each single year of age between 55 and 64, and five socioeconomic status strata (based on median household income and distribution of education of adults living in Los Angeles County during the 1980 census). The exact age match resulted in exclusion of 101 control subjects younger than 55 years, and 152 control subjects older than 64 years who originally were within 36 months of the age of their corresponding case but outside the restricted age range. An additional 42 case patients and 12 control subjects were excluded because they fell into age and socioeconomic strata that had only cases or only controls. The analyses are based on 1123 case patients (100 of whom had in situ disease) and 904 control subjects.

We estimated odds ratios (OR), 95% confidence intervals (CI) for the odds ratio (based on the standard error of the log odds), and tests for linear trend across ordinal values of categorical variables by conditional logistic regression methods. All reported $P$-values are two-sided. Heterogeneity of trends was evaluated using a likelihood ratio test.

### RESULTS

Table 1 presents distributions and unadjusted OR for potential confounding factors of the association between physical exercise activity and breast cancer risk. Breast cancer risk increased with increasing levels of Quetelet's index at reference date (trend $P < 0.001$), but was unrelated to Quetelet's index at age 18. Women who gained excessive weight between age 18 and reference age were at greater risk of breast cancer than those whose weight was stable. Quetelet's index at reference age was highly correlated with per cent weight change between age 18 and reference age (Pearson correlation, $r = 0.75$). OR estimates for the extreme categories of the two variables were similar.

Both family history of breast cancer and age at first term pregnancy among parous women were positively associated with breast cancer risk (Table 1). Age at menarche and age at menopause were not associated with risk. We included Quetelet's index at reference date, ages at first full-term pregnancy, menarche and menopause, family history, and interviewer in all multivariate models assessing the relationship between physical exercise activity and breast cancer risk (Tables 2, 3 and 4). Duration of use of hormone replacement therapy was not related to any exercise activity variable and was not included in the multivariate models.

Neither average MET-hours per week of physical exercise activity in which a woman engaged between menarche and age 39 years, nor that in which she engaged from age 40 years to the reference age was related to breast cancer risk (Table 2). Risk estimates were similar to those shown in Table 2 when both measures were included in the same model. However, breast cancer risk was substantially lower among women who maintained a high level of exercise activity (averaging at least 17.6 MET-hours per week)

© 1999 Cancer Research Campaign

**Table 1** Odds ratios (OR) and 95% confidence intervals (CI) for potential confounding factors of the association between physical activity and breast cancer risk among post-menopausal women aged 55–64 years

| Variable | No. of cases | No. of controls | OR | (95% CI) | Trend *P*-value |
|---|---|---|---|---|---|
| Quetelet's index[a] at reference date[b] | | | | | |
| <21.7 | 232 | 233 | 1.00 | – | |
| 21.7–23.6 | 256 | 219 | 1.17 | (0.90–1.53) | |
| 23.7–27.0 | 284 | 225 | 1.31 | (1.01–1.71) | |
| ≥ 27.1 | 351 | 227 | 1.58 | (1.22–2.04) | < 0.001 |
| Quetelet's index[a] at age 18 | | | | | |
| < 18.9 | 317 | 228 | 1.00 | – | |
| 19.0–20.29 | 233 | 227 | 0.75 | (0.58–0.97) | |
| 20.3–22.16 | 318 | 224 | 1.05 | (0.82–1.35) | |
| ≥ 22.17 | 255 | 225 | 0.80 | (0.62–1.03) | 0.41 |
| Percent change in weight from age 18 to reference date | | | | | |
| Negative change to no change | 141 | 140 | 1.00 | – | |
| 0.1–16.9% | 359 | 308 | 1.11 | (0.83–1.49) | |
| 17.0–29.1% | 251 | 203 | 1.18 | (0.87–1.61) | |
| ≥ 29.2% | 372 | 253 | 1.47 | (1.09–1.97) | 0.005 |
| Age at menarche | | | | | |
| < 12 | 224 | 175 | 1.00 | – | |
| 12 | 298 | 256 | 0.86 | (0.65–1.12) | |
| 13 | 332 | 261 | 0.96 | (0.73–1.25) | |
| ≥ 14 | 269 | 212 | 0.93 | (0.70–1.22) | 0.90 |
| Age at menopause | | | | | |
| < 40 | 61 | 53 | 1.00 | – | |
| 40–44 | 142 | 111 | 1.11 | (0.70–1.75) | |
| 45–49 | 311 | 277 | 1.00 | (0.66–1.52) | |
| 50–54 | 495 | 373 | 1.22 | (0.81–1.84) | |
| ≥ 55 | 114 | 90 | 1.17 | (0.72–1.88) | 0.21 |
| Age at first full-term pregnancy | | | | | |
| < 20 | 139 | 116 | 1.15 | (0.86–1.54) | |
| 20–24 | 426 | 421 | 1.00 | – | |
| 25–29 | 245 | 193 | 1.25 | (0.98–1.59) | |
| 30–34 | 88 | 57 | 1.53 | (1.05–2.23) | |
| ≥ 35 | 38 | 21 | 1.69 | (0.96–2.99) | 0.004[c] |
| Nulliparous | 187 | 96 | 1.82 | (1.36–2.44) | |
| First-degree family history of breast cancer | | | | | |
| No | 907 | 798 | 1.00 | – | |
| Yes | 208 | 100 | 1.84 | (1.41–2.39) | |
| Don't know/adopted | 8 | 6 | 1.62 | (0.54–4.83) | |

[a]Weight (kg)/height$^2$ (m); [b]one year prior to breast cancer diagnosis for cases and corresponding date for controls; [c]trend test for parous women.

throughout their lifetimes, from menarche to reference date (OR = 0.55; 95% CI 0.37–0.83), compared to those with lifelong inactivity (Table 3).

Exercise during the first 10 years after menarche was not associated with breast cancer risk (Table 2). Likewise, average MET-hours of exercise per week in the 10-year period preceding the reference date was not associated with risk (trend $P = 0.32$), although women who averaged at least 24.5 MET-hours per week had a modestly reduced risk (OR = 0.71; 95% CI 0.48–1.07).

Total years in which women exercised more than 4 h per week (Table 3) predicted breast cancer risk (trend $P = 0.01$). Women who maintained this activity level for 12 or more years were 29% less likely to develop post-menopausal breast cancer than women who never exercised at that level.

We examined the effects of exercise activity among women with weight change above and below the median value of controls (17% increase in weight) in a single logistic regression model to evaluate effect modification (Table 4). We included Quetelet's index at reference data as a continuous term in these analyses. Within the group of women who maintained their weight (< 17% increase), three measures of exercise were associated with reduced breast cancer risk, while none was associated with risk among women with greater weight gain. Among women with stable weight, those who exercised at least 17.6 MET-hours per week throughout their lifetimes reduced their breast cancer risk by more than 55% relative to women who were inactive in both age periods. Among women who maintained their weight, breast cancer risk declined with increasing number of years the woman exercised more than 4 h per week (trend $P = 0.009$); with risk reduced more than 40% for those exercising at this level for at least 12 years since menarche. Risk was reduced 48% for those who averaged at least 24.5 MET-hours over the 10 years preceding the reference date. Trends in risk for MET-hours of activity within 10 years prior to reference age ($P = 0.06$) and trends in risk for lifetime MET-hours of activity ($P = 0.005$) differed between women with a sizable weight gain (≥ 17%) and women who maintained stable weight (< 17% gain). Trends in risk for years of activity that averaged more than 4 h per week did not differ between the two weight gain groups ($P = 0.30$).

### DISCUSSION

Most risk factors for female breast cancer can be understood as measures of cumulative exposure of the breast to oestrogens and progesterone. Women with early menarche have greater breast cancer risk than those with later menarche and this risk factor contributes substantially to overall breast cancer risk in young women (Kelsey et al, 1993). Early menarche represents more years of exposure to ovarian hormones because it predicts a more rapid onset of regular ovulatory menstrual cycles during adolescence and higher circulating oestrogen levels later in reproductive life (Vihko and Apter, 1984; Apter et al, 1989). Women who experience earlier menopause have lower breast cancer risk than those who stop menstruating later (Kelsey et al, 1993). Weight gain as an adult and post-menopausal obesity increase breast cancer risk after the menopause (Hunter and Willett, 1993; Huang et al, 1997); this is likely due to greater oestrogen exposure in obese than in thinner post-menopausal women (Key and Pike, 1988; Potischman et al, 1996; Thomas et al, 1997) because of the high levels of oestrone production occurring in adipose tissue (MacDonald et al, 1978; Kirschner et al, 1981).

Based on our understanding of the importance of ovarian hormones to breast cancer risk, we hypothesized that regular participation in exercise activities sufficient to alter menstrual cycle patterns and ovulatory status during reproductive years should reduce breast cancer risk (Bernstein et al, 1992). Exercise may reduce a woman's cumulative exposure to oestrogen by delaying menarche (Frisch et al, 1980, 1981), lowering levels of serum oestrogen (Russell et al, 1984; Broocks et al, 1990), and increasing the frequency of anovulation (Russell et al, 1984; Bernstein et al, 1987). During a woman's perimenopausal and post-menopausal years, exercise may reduce hormonal exposure through weight maintenance.

In our previous case-control study of women aged 40 or younger, we examined lifetime exercise patterns and observed a sizable reduction in breast cancer risk among women who

 *© 1999 Cancer Research Campaign*

**Table 2** Odds ratios (OR) and 95% confidence intervals (CI) for the association between time-specific measures of physical exercise activity and breast cancer risk among post-menopausal women aged 55–64 years

| Time period of exercise activity / Category of activity | No. of cases | No. of controls | OR | Adjusted OR[a] | 95% CI | Trend *P*-value |
|---|---|---|---|---|---|---|
| First 10 years after menarche[b] (average MET-hours[c] per week) | | | | | | |
| No activity | 829 | 664 | 1.00 | 1.00 | – | |
| 0.1–9.8 | 118 | 81 | 1.12 | 1.10 | (0.80–1.52) | |
| 9.9–16.5 | 65 | 70 | 0.76 | 0.75 | (0.51–1.10) | |
| ≥ 16.6 | 111 | 89 | 1.00 | 0.92 | (0.61–1.40) | 0.43 |
| From menarche to age 39[d] (average MET-hours per week) | | | | | | |
| No activity | 740 | 571 | 1.00 | 1.00 | – | |
| 0.1–3.74 | 85 | 102 | 0.64 | 0.67 | (0.48–0.93) | |
| 3.75–8.74 | 118 | 89 | 1.05 | 1.09 | (0.79–1.51) | |
| 8.75–17.59 | 107 | 79 | 1.07 | 1.05 | (0.75–1.48) | |
| ≥ 17.6 | 73 | 63 | 0.90 | 0.85 | (0.57–1.28) | 0.79 |
| From age 40 to reference date[ef] (average MET-hours per week) | | | | | | |
| No activity | 756 | 577 | 1.00 | 1.00 | – | |
| 0.1–3.74 | 81 | 75 | 0.84 | 0.86 | (0.60–1.23) | |
| 3.75–8.74 | 86 | 70 | 0.94 | 0.92 | (0.64–1.31) | |
| 8.75–17.59 | 112 | 84 | 1.05 | 1.07 | (0.77–1.49) | |
| ≥ 17.6 | 88 | 98 | 0.73 | 0.81 | (0.57–1.15) | 0.46 |
| From 10 years prior to reference date until reference date[g] (average MET-hours per week) | | | | | | |
| No activity | 792 | 605 | 1.00 | 1.00 | – | |
| 0.01–6.9 | 81 | 71 | 0.85 | 0.87 | (0.61–1.25) | |
| 7.0–13.9 | 89 | 77 | 0.93 | 0.92 | (0.65–1.31) | |
| 14.0–24.4 | 98 | 75 | 1.01 | 1.09 | (0.76–1.55) | |
| ≥ 24.5 | 63 | 76 | 0.64 | 0.71 | (0.48–1.07) | 0.32 |

[a]Odds ratio adjusted for categories of body-mass index (Quetelet's index) at reference date, age at first full-term pregnancy, family history of breast cancer, age at menarche, age at menopause, and interviewer. [b]Adjusted model includes continuous term for average MET-hours from 10 years after menarche until the reference date. [c]Product between metabolic equivalent of energy expenditure and hours of activity. [d]Adjusted model includes continuous term for average MET-hours per week of exercise from age 40 to reference date. [e]One year prior to breast cancer diagnosis for the cases, and corresponding date for controls. [f]Adjusted model also includes continuous term for average MET-hours from menarche to age 39. [g]Adjusted model also includes continuous term for average MET-hours of activity up to 10 years prior to reference date.

**Table 3** Odds ratios (OR) and 95% confidence intervals (CI) for the association between lifetime measures of physical exercise activity and breast cancer risk among post-menopausal women aged 55–64 years

| Measure of lifetime activity / Category of activity | No. of cases | No. of controls | OR | Adjusted OR[a] | 95% CI | Trend *P*-value |
|---|---|---|---|---|---|---|
| Exercise activity from menarche to the reference date[b] (average MET-hours[c] per week) | | | | | | |
| No activity | 575 | 427 | 1.00 | 1.00 | – | |
| 0.1–< 17.59 | 492 | 404 | 0.90 | 0.88 | (0.72–1.07) | |
| ≥ 17.6 | 56 | 73 | 0.59 | 0.55 | (0.37–0.83) | 0.01 |
| Years engaged in more than 4 h per week of exercise activity[d] | | | | | | |
| 0 | 811 | 616 | 1.00 | 1.00 | – | |
| 1–11 | 196 | 171 | 0.86 | 0.83 | (0.65–1.06) | |
| 12 | 116 | 117 | 0.74 | 0.71 | (0.52–0.96) | 0.01 |

[a]Odds ratio adjusted for categorical terms for body-mass index (Quetelet's index) at reference date, age at first full-term pregnancy, family history of breast cancer, age at menarche, age at menopause, and interviewer. [b]One year prior to breast cancer diagnosis for the cases, and corresponding date for controls. [c]Product between metabolic equivalent of energy expenditure and hours of activity. [d]Adjusted model also includes continuous term for number of years in which the average amount of exercise was 4 h per week or less.

© 1999 Cancer Research Campaign

**Table 4** Odds ratios (OR) and 95% confidence intervals (CI) for association between measures of physical exercise activity and breast cancer risk among post-menopausal women aged 55–64 years by percent adult weight change.

| | Per cent weight change between age 18 and reference date[a] | | | | | | | |
|---|---|---|---|---|---|---|---|---|
| **Time period of Exercise activity** | **< 17.0%** | | | | **≥ 17.0%** | | | |
| **Category of activity** | **Cases/ Controls** | **OR[b]** | **95% CI** | **Trend *P*-value** | **Cases/ Controls** | **OR[b]** | **95% CI** | **Trend *P*-value** |
| From menarche to to reference date (average MET-hours[c] per week) | | | | | | | | |
| No activity | 214/197 | 1.00 | – | | 334/230 | 1.00 | – | |
| 0.1–< 17.59 | 237/211 | 0.81 | (0.63–1.04) | | 255/193 | 0.95 | (0.74–1.21) | |
| ≥ 17.6 | 22/42 | 0.42 | (0.24–0.75) | 0.003 | 34/31 | 0.70 | (0.40–1.19) | 0.26 |
| Number of years engaged in more than 4 h of exercise activity per week[d] | | | | | | | | |
| 0 | 356/301 | 1.00 | – | | 455/315 | 1.00 | – | |
| 1–11 | 90/75 | 0.84 | (0.59–1.20) | | 106/96 | 0.82 | (0.60–1.13) | |
| ≥ 12 | 54/74 | 0.59 | (0.40–0.88) | 0.009 | 62/43 | 0.86 | (0.56–1.33) | 0.25 |
| From 10 years prior to Reference age until reference date (average MET-hours per week) | | | | | | | | |
| 0 | 332/272 | 1.00 | – | | 460/333 | 1.00 | – | |
| 0.1–6.9 | 41/40 | 0.80 | (0.49–1.28) | | 40/31 | 0.94 | (0.56–1.58) | |
| 7.0–13.9 | 42/38 | 0.80 | (0.49–1.31) | | 47/39 | 1.02 | (0.64–1.63) | |
| 14.0–24.4 | 49/43 | 1.05 | (0.66–1.68) | | 49/32 | 1.05 | (0.64–1.72) | |
| ≥ 24.5 | 36/57 | 0.52 | (0.32–0.85) | 0.04 | 27/19 | 1.12 | (0.59–2.15) | 0.75 |

[a]One year prior to breast cancer diagnosis for cases and corresponding date for controls. [b]Adjusted for categories of age at first full-term pregnancy, family history of breast cancer, age at menarche, age at menopause, interviewer, and a continuous term for Quetelet's index at reference date. [c]Product between metabolic equivalent of energy expenditure and hours of exercise activity. [d]Adusted model also includes continuous term for number of years in which the average amount of exercise was 4 h per week or less. [e]Adjusted model also includes continuous term for average MET-hours per week of exercise up to 10 years prior to reference date.

averaged at least 3.8 h per week of exercise during their reproductive years (Bernstein et al, 1994). These results led us to pursue whether exercise might affect breast cancer risk among post-menopausal women.

Measurement of lifetime exercise in this study has enabled us to examine activity patterns in different ways. We summarized lifetime physical activity into two time periods and evaluated whether exercise during the reproductive years might affect risk differently than exercise in the perimenopausal and post-menopausal years. We also evaluated lifetime activity. Breast cancer risk was appreciably reduced among women who exercised at high levels throughout their lifetime. Although activity in the first 10 years after menarche was related to reduced breast cancer risk in our study of younger women (Bernstein et al, 1994), we did not find this effect in the present study of post-menopausal women aged 55–64 years, unless women continued regular exercise.

A high exercise level during the 10-year perimenopausal and early post-menopausal period was moderately protective compared to inactivity during those years, but the overall association was non-linear. Years that women exercised more than 4 h per week, on the other hand, was strongly protective with a clear dose–response relationship.

Weight gain in adulthood is generally associated with an increased risk of post-menopausal breast cancer (Ballard-Barbash et al, 1990; Brinton and Swanson, 1992; Barnes-Josiah et al, 1995; Ziegler et al, 1996; Huang et al, 1997). Post-menopausal women who exercise have lower circulating levels of oestrone (Nelson et al, 1988; Cauley et al, 1989), and this relationship appears to be independent of body mass (Cauley et al, 1989). The effects of exercise activity in the present study varied according to amount of weight gained during adulthood. Exercise was strongly associated with reduced post-menopausal breast cancer risk among women whose weight gain during adulthood was minimal, but was not clearly associated with breast cancer risk among women who gained greater amounts of weight.

Two recent studies of physical activity and breast cancer risk also examined differences in risk reduction according to body mass (Coogan et al, 1997; Thune et al, 1997), with results generally consistent with data reported here. Occupational physical activity was mildly protective for breast cancer among post-menopausal women in the study of Coogan et al (1997), but among lean women the protective effect was stronger. In a large prospective study of Norwegian women, an inverse association between the highest level of a self-rated activity score and breast cancer risk was observed with the most sizable reduction in risk among women with a lean body-mass (Thune et al, 1997).

Although correlated, weight change during adulthood and obesity represent somewhat different variables. To account for potential residual confounding from obesity, we adjusted for body-mass index at the reference age in all analyses that evaluated the effects of exercise and weight change. We further examined average body-mass index values at the reference age in each category of the activity variables within each weight gain category. We found only slight differences between case patients and control subjects.

Results from the present study emphasize the complexity of the relationship between exercise activity and post-menopausal breast cancer risk. Early exercise activity, if not sustained, does not appear to affect post-menopausal breast cancer risk. However, lifetime exercise when measured by average MET-hours per week

 *© 1999 Cancer Research Campaign*

or by exercise that averages more than 4 h per week, as well as vigorous exercise of sizable duration during the perimenopausal and early post-menopausal years are moderately associated with reduced breast cancer risk. Such exercise, especially when combined with maintenance of a relatively stable weight during adulthood, substantially reduces the risk of post-menopausal breast cancer.

Our results appear to suggest that among women who maintain a lean body, exercise exerts a separate, independent effect on breast cancer risk. However, our body-mass measure does not take into account the relative amounts of muscle mass and body fat, which, even among women with relatively stable weight, may vary according to activity level. As a consequence, the protective effect of exercise may not be independent of the amount of adipose tissue. Future studies are needed to identify whether exercise affects post-menopausal breast cancer risk by reducing the amount of adipose tissue, by independently lowering oestrogen levels, or by affecting other less studied factors such as insulin resistance.

### ACKNOWLEDGEMENTS

Funding for the study was provided by grant CA17054 from the National Cancer Institute and the LK Whittier Foundation, a private foundation supporting biomedical research. Dr Carpenter was supported by the State of California Breast Cancer Research Program, 1FB0202, and a supplemental fellowship awarded by the USC/Norris Comprehensive Cancer Center. Cancer incidence data have been collected under Subcontract 050L-8709-S1149 with the Contractor, Public Health Institute. The subcontract is supported by the California Department of Health Services as part of its statewide cancer-reporting program mandated by Health and Safety Code Sections 103875 and 103885. The ideas and opinions expressed herein are those of the authors and no endorsement by the State of California, Department of Health Services, or the Contractor is intended or should be inferred.

### REFERENCES

Ainsworth BE, Haskell WL, Leon AS, Jacobs Jr DR, Montoye HJ, Sallis JF and Paffenbarger RS Jr (1993) Compendium of physical activities: classification of energy costs of human physical activities. *Med Sci Sports Exerc* **25**: 71–80

Albanes D, Blair A and Taylor PR (1989) Physical activity and risk of cancer in the NHANES I population. *Am J Publ Health* **79**: 744–750

Apter D, Reinila M and Vihko R (1989) Some endocrine characteristics of early menarche, a risk factor for breast cancer, are preserved into adulthood. *Int J Cancer* **44**: 783–787

Ballard-Barbash R, Schatzkin A, Taylor PR and Kahle LL (1990) Association of change in body mass with breast cancer. *Cancer Res* **50**: 2152–2155

Barnes-Josiah D, Potter JD, Sellers TA and Himes JH (1995) Early body size and subsequent weight gain as predictors of breast cancer incidence (Iowa, United States). *Cancer Causes Control* **6**: 112–118

Bernstein L, Ross RK, Lobo RA, Hanisch R, Krailo MD and Henderson BE (1987) The effects of moderate physical activity on menstrual cycle patterns in adolescence: implications for breast cancer prevention. *Br J Cancer* **55**: 681–685

Bernstein L, Ross RK and Henderson BE (1992) Prospects for the primary prevention of breast cancer. *Am J Epidemiol* **135**: 142–152

Bernstein L, Henderson BE, Hanisch R, Sullivan-Halley J and Ross RK (1994) Physical exercise and reduced risk of breast cancer in young women. *J Natl Cancer Inst* **86**: 1403–1408

Brinton LA and Swanson CA (1992) Height and weight at various ages and risk of breast cancer. *Ann Epidemiol* **2**: 597–609

Broocks A, Pirke KM, Schweiger U, Tuschl RJ, Laessle RG, Strowitzki T, Horl E, Horl T, Haas W and Jeschke D (1990) Cyclic ovarian function in recreational athletes. *J Appl Physiol* **68**: 2083–2086

Cauley JA, Gutai JP, Kuller LH, LeDonne D and Powell JG (1989) The epidemiology of serum sex hormones in postmenopausal women. *Am J Epidemiol* **129**: 1120–1131

Chen C-L, White E, Malone KE and Daling JR (1997) Leisure-time physical activity in relation to breast cancer among young women (Washington, United States). *Cancer Causes Control* **8**: 77–84.

Coogan PF, Newcomb PA, Clapp RW, Trentham-Dietz A, Baron JA and Longnecker MP (1997) Physical activity in usual occupation and risk of breast cancer (United States). *Cancer Causes Control* **8**: 626–631

D'Avanzo B, Nanni O, La Vecchia C, Franceschi S, Negri E, Giacosa A, Conti E, Montella M, Talamini R and Decarli A (1996) Physical activity and breast cancer risk. *Cancer Epidemiol Biomarkers Prev* **5**: 155–160

Dorgan JF, Brown C, Barrett M, Splanski GL, Kreger BE, D'Agostino RB, Albanes D and Schatzkin A (1994) Physical activity and risk of breast cancer in the Framingham Heart Study. *Am J Epidemiol* **139**: 662–669

Dosemeci M, Hayes RB, Vetter R, Hoover RN, Tucker M, Engin K, Unsal M and Blair A (1993) Occupational physical activity, socioeconomic status, and risks of 15 cancer sites in Turkey. *Cancer Causes Control* **4**: 313–321

Frisch RE, Wyshak G and Vincent L (1980) Delayed menarche and amenorrhea in ballet dancers. *N Engl J Med* **303**: 17–19

Frisch RE, Gotz-Welbergen AV, McArthur JW, Albright T, Witschi J, Bullen B, Birnholz J, Reed RB and Hermann H (1981) Delayed menarche and amenorrhea of college athletes in relation to age of onset of training. *JAMA* **246**: 1559–1563

Frisch RE, Wyshak G, Albright NL, Albright TE, Schiff I, Jones KP, Witschi J, Shaing E, Koff E and Marguglio M (1985) Lower prevalence of breast cancer and cancers of the reproductive system among former college atheletes compared to non-athletes. *Br J Cancer* **52**: 885–891.

Gammon MD, Schoenberg JB, Britton JA, Kelsey JL, Coates RJ, Brogan D, Potischman N, Swanson CA, Daling JR, Standford JL and Brinton LA (1998) Recreational physical activity and breast cancer risk among women under 45 years. *Am J Epidemiol* **147**: 273–280

Huang Z, Hankinson SE, Colditz GA, Stampfer MJ, Hunter DJ, Manson JE, Hennekens CH, Rosner B, Speizer FE and Willett WC (1997) Dual effects of weight and weight gain on breast cancer risk. *JAMA* **278**: 1407–1411

Hunter DJ and Willett WC (1993) Diet, body size, and breast cancer. *Epidemiol Rev* **15**: 110–132

Kelsey JL, Gammon MD and John EM (1993) Reproductive factors and breast cancer. *Epidemiol Rev* **15**: 36–47.

Key TJ and Pike MC (1988) The role of oestrogens and progestagens in the epidemiology and prevention of breast cancer. *Eur J Cancer Clin Oncol* **24**: 29–43.

Kirschner MA, Ertel N and Schneider G (1981) Obesity, hormones, and cancer. *Cancer Res* **41**: 3711–3717

MacDonald PC, Edman CD, Hemsell DL, Porter JC and Siiteri PK (1978) Effect of obesity on conversion of plasma androstenedione to estrone in postmenopausal women with and without endometrial cancer. *Am J Obstet Gynecol* **130**: 448–455

McTiernan A, Stanford JL, Weiss NS, Daling JR and Voigt LF (1996) Occurrence of breast cancer in relation to recreational exercise in women aged 50–64 years. *Epidemiology* **77**: 598–604

Mezzetti M, La Vecchia C, Decarli A, Boyle P, Talamini R and Franceschi S (1998) Population attributable risk for breast cancer: Diet, nutrition, and physical exercise. *J Natl Cancer Inst* **90**: 389–394

Mittendorf R, Longnecker MP, Newcomb PA, Dietz AT, Greenberg ER, Bogdan GF, Clapp RW and Willett WC (1995) Strenuous physical activity in young adulthood and risk of breast cancer (United States). *Cancer Causes Control* **6**: 347–353

Nelson ME, Meredith CN, Dawson-Hughes B and Evans WJ (1988) Hormone and bone mineral status in endurance-trained and sedentary postmenopausal women. *J Clin Endocrinol Metab* **66**: 927–933

Potischman N, Swanson CA, Siiteri P and Hoover RN (1996) Reversal of relation between body mass and endogenous estrogen concentrations with menopausal status. *J Natl Cancer Inst* **88**: 756–758

Rockhill B, Willett WC, Hunter DJ, Manson JE, Hankinson SE, Spiegelman D and Colditz GA (1998) Physical activity and breast cancer risk in a cohort of young women. *J Natl Cancer Inst* **90**: 1155–1160

Russell JB, Mitchell D, Musey PI and Collins DC (1984) The relationship of exercise to anovulatory cycles in female athletes: hormonal and physical characteristics. *Obstet Gynecol* **63**: 452–456

Thomas HV, Key TJ, Allen DS, Moore JW, Dowsett M, Fentiman IS and Wang DY (1997) Re: Reversal of relation between body mass and endogenous estrogen concentrations with menopausal status. *J Natl Cancer Inst* **89**: 396–398

© 1999 Cancer Research Campaign

Thune I, Brenn T, Lund E and Gaard M (1997) Physical activity and the risk of breast cancer. *N Engl J Med* **336**: 1269–1275

Vihko R and Apter D (1984) Endocrine characteristics of adolescent menstrual cycles: impact of early menarche. *J Steroid Biochem* **20**: 231–236

Vihko VJ, Apter DL, Pukkula EI, Oinonen MT, Hakulinen TR and Vihko RK (1992) Risk of breast cancer among female teachers of physical education and languages. *Acta Oncol* **31**: 201–204

Williamson DF, Madans J, Anda RF, Kleinman JC, Kahn HS and Byers T (1993) Recreational physical activity and ten-year weight change in a US national cohort. *Int J Obesity* **17**: 279–286

Ziegler RG, Hoover RN, Nomura AMY, West DW, Wu AH, Pike MC, Lake AJ, Horn-Ross PL, Kolonel LN, Siiteri PK and Fraumeni JF Jr (1996) Relative weight, weight change, height, and breast cancer risk in Asian-American women. *J Natl Cancer Inst* **88**: 650–660

*© 1999 Cancer Research Campaign*
